# RGBD-Inertial Trajectory Estimation and Mapping for Ground Robots

**DOI:** 10.3390/s19102251

**Published:** 2019-05-15

**Authors:** Zeyong Shan, Ruijian Li, Sören Schwertfeger

**Affiliations:** 1 School of Information Science & Technology, ShanghaiTech University, Shanghai 201210, China; lirj@shanghaitech.edu.cn; 2 Chinese Academy of Sciences, Shanghai Institute of Microsyst & Information Technology, Shanghai 200050, China; 3 University of Chinese Academy of Sciences, Beijing 100049, China

**Keywords:** visual-inertial systems, SLAM, inertial motion tracking, ground robots, rescue robots, sensor fusion, state estimation, RGBD sensor

## Abstract

Using camera sensors for ground robot Simultaneous Localization and Mapping (SLAM) has many benefits over laser-based approaches, such as the low cost and higher robustness. RGBD sensors promise the best of both worlds: dense data from cameras with depth information. This paper proposes to fuse RGBD and IMU data for a visual SLAM system, called VINS-RGBD, that is built upon the open source VINS-Mono software. The paper analyses the VINS approach and highlights the observability problems. Then, we extend the VINS-Mono system to make use of the depth data during the initialization process as well as during the VIO (Visual Inertial Odometry) phase. Furthermore, we integrate a mapping system based on subsampled depth data and octree filtering to achieve real-time mapping, including loop closing. We provide the software as well as datasets for evaluation. Our extensive experiments are performed with hand-held, wheeled and tracked robots in different environments. We show that ORB-SLAM2 fails for our application and see that our VINS-RGBD approach is superior to VINS-Mono.

## 1. Introduction

With the sensor and algorithm innovations [1], mobile robots are getting smaller and smarter and are addressing new applications in medicine, agriculture, and security applications [2,3]. The improved sensors allow them to become much more capable for perception, mapping, and navigation.

Unlike indoor navigation, which focuses more on 2D, even and structured environments, rescue robots need more mobility to face various, complicated scenarios such as slopes, stairs, and tunnels [4,5,6]. Search and rescue robots are usually equipped with LIDAR and high-quality IMU sensors, which are fused with the wheel odometry to estimate the poses and the map [7,8,9]. However, these leads to expensive robots, which is undesirable, because such mobile rescue robots have a high risk of breaking or being lost during operation. In the past years, we have seen great advances in the area of computer vision, also with respect to vSLAM (visual Simultaneous Localization and Mapping) [10]. Since then, many methods have been proposed to improve the accuracy, robustness, and efficiency of vSLAM, such as using first estimation Jacobian [11] to reduce the inherent nonlinearity in the system. Optimization-based methods also have become very popular [12]. Additionally, monocular cameras were combined with other sensors such as depth cameras [13,14] and IMU [15,16,17] to achieve more robust performance.

However, these approaches are mostly used in hand-held or UAV (Unmanned Aerial Vehicle) applications, where the motions follow smooth, curved trajectories and frequently change the velocity. Thus, even with strong rotation, when there are few features to track in the image sequences, the IMU can still sense the motion well, which, thus, limits the pose estimation to an acceptable error [15,17].

On the other hand, ground robots have some unique advantages in rescue scenarios [18], compared to aerial robots, for example the long operation time, the rich sensor payload [19] and the ability to traverse in very confined areas. Hence, ground robots still play a critical role in search and rescue scenarios [6]. When rescue robots traverse difficult 3D terrain, the motions can be more complicated [4,20] than those of a UAV. There are very sudden accelerations when falling off a small edge or a stair step, or bumping into a small obstacle. This will result in strong ego-motions, which make it difficult for vSLAM and VIO (Visual Inertial Odometry) to track the features. Fusing the vision data with IMU readings is a direct idea to solve this problem. However, moving straight on a planar environment, the trajectories are mostly in constant acceleration and quite often constant in speed, which leads to a close to zero acceleration. In this case the VINS (Vision-aided INertial System) model suffers extra unobservable directions [21], which leads to inaccurate estimations.

This challenge becomes even tougher for tracked robots or small-sized robots. Besides above problem of unobservability, the IMU readings suffer from large amounts of noise. This is because the small, tracked robots, with a relatively small mass, get easily excited from small obstacles such as stones or sand, over which the robot drives, leading to big and frequent vibrations. An additional challenge rescue robots face is, that there are often high illumination changes and also, that environments can have few features, because everything is covered with dust [22]. So visual trackers for rescue robots face additional challenges.

In recent decades we have seen many innovative and improved sensor concepts, such as RGBD cameras, LIDAR and event cameras [23]. They are perceiving the environments using different approaches and integrate several different sensors on a single board thanks to the advances in MEMS (MicroElectroMechanical System) technology. Kinect and Kinect V2 are two very popular RGBD cameras using structured light and the latter ToF (Time of Flight) technology, respectively. They are both quite large and do not work outdoors, because the sunlight is over shining the infrared light emitted by the devices. LIDARs work outdoors and are quite accurate, but they deliver quite sparse data, are quite expensive and relatively fragile, due to the moving parts (mirror). In the last few years, Intel released their RealSense camera series [24], from R200 to ZR300 to D415/D435 [25] and other models. Some of them share the same market niche as Kinect, while others are facing robotic systems which are compact and relatively cheap. A big advantage of some of those devices, such as ZR300, is, that they come with an integrated IMU. For robotics, a challenging problem is the limited measurement range of those sensors, with a large minimum range and a small maximum. This significantly limits its application and thus many open source VIO or SLAM systems [15,17] only make use of the integrated fisheye camera and IMU sensor. Fortunately, Intel released the D435i [26] after the D415/D435. It integrates the same IMU as the ZR300, but uses the vision sensors of the D435, such that we can take advantage of D435’s excellent range (0.2–10 m+), combined with the time-synchronized accelerometer and gyroscope measurements. Another advantage of the Intel RealSense sensor series is, that they also work outdoors in sunny conditions, due to their stereo-infrared camera approach [24,27,28], but also project an infrared pattern to be able to detect features-less surfaces.

The goal of our work is to enable a small rescue robot to do visual SLAM and thus ultimately navigation. We address the difficulties described above and propose to utilize the three different sensor readings of color, depth and IMU provided by the Intel RealSense D435i camera. For that, we base our solution, VINS-RGBD, on the open source VINS implementation [15], which is using RGB and IMU. We extend the framework to also incorporate the depth readings and ultimately build an RGBD inertia SLAM system for ground robots. The main contributions of our work are as follows:

- Formulation and implementation of a depth-integrated initialization process for the VINS-RGBD system.
- Formulation and implementation of a depth-integrated Visual Inertial Odometry (VIO), overcoming the degenerated cases of a vision and IMU only VIO system.
- Design and implementation of a backend mapping function to build dense point clouds with noise suppression, which is suitable for further map post processing and path planning.
- A color-depth-inertial dataset with handheld, wheeled robot, and tracked robot motion, with tracking system data for ground truth poses.
- Thorough evaluation of the proposed VINS-RGBD system using the three datasets mentioned above.

We provide the dataset [29], which also includes a video of the experiments, and VINS-RGBD [30] as open source for the benefit of the robotics and visual SLAM communities.

The rest of the paper is structured as follows: Section 2 describes important related work and the applicability towards VIO with ground robots. Section 3 gives an overview of the system which we base our work, VINS-Mono, and the observability problem it meets. Section 4 introduces our VINS-RGBD approach while Section 5 gives corresponding experiments and results. In Section 6 we draw conclusions and discuss future work.

## 2. Related Work

Compared to LIDAR Simultaneous Localization and Mapping (SLAM), which has under development for decades, visual SLAM (vSLAM) has recently gained more and more attention, especially after the influential work of Davison et al. [10], which showed that SLAM with a single camera can run in real-time on a PC. Extended Kalman Filter (EKF) or its variants are often used in LIDAR SLAM, and also in that first MonoSLAM [10]. Optimization based methods are becoming more popular, since they can find the sparsity of the Hessian matrix in the optimization function and can usually provide more accurate results. PTAM [31] was the first optimization based method and also the first one to propose to do tracking and mapping in parallel. Based on that tracking front end and optimization back end, approaches such as ORB-SLAM [12] were developed, which additionally use bag-of-words [32] to do loop detection to reduce the accumulative error. After projecting the environment to image planes, the depth information is lost and only the perspective geometry relationship is saved. Therefore, monocular SLAM is up-to-scale. However, combined with another camera with a fixed, known baseline, we can obtain the depth by calculating the disparity of sufficiently distinguishable feature points. Next to this stereo camera approach also structured light and Time of Flight (ToF) techniques, combined with a color camera, can also provide depth and color data, thus RGBD data. Visual SLAM with RGBD cameras [13,33] can be applied to robot autonomy and navigation. As both stereo and RGBD SLAM are using a single camera as the perception source, the performance is limited by camera characteristics such as motion blur under fast rotation, distortion caused by the rolling shutter, and also the short measurement range of the RGBD camera.

For ground robots quite often the trajectory estimation, so the localization of the robot, is assumed to be on the flat ground. We can thus estimate the trajectory under the 2D plane constraint, which accelerates the estimation speed and also improves the robustness with the motion model constraint. Scaramuzza [34] models the 2D motion to approximate planar circular motion, thus a 1-point minimum relative pose estimate solver can be applied. With a general planer motion model, an iterative 2-point algorithm [35] using the Newton method, a 3-point algorithm which is using a linear equation and non-iterative 2-point algorithms [36] were proposed.

Unlike Unmanned Ground Vehicles (UGVs), Unmanned Aerial Vehicles (UAVs), especially quad-copters, have to estimate their motion in 3D with 6 degrees of freedom. To avoid obstacles also the unknown scale factor has to be solved. So there is considerable work on vSLAM for UAVs where the cameras are combined with inertial measurement units (IMU) to achieve robust and scale deterministic pose estimation. Due to the complex motions of the UAV, the IMU is sufficiently activated and thus unobservable states are avoided. The visual and inertial fusion also called VINS (Visual INertial System). It is usually divided into the categories of loosely and tightly coupled systems, where the former [37,38] treats the two sensors as individual pose estimation sources and fuses them by using EKF. Tight coupled visual inertial fusion has both EKF based algorithms such as ROVIO [17], MSCKF [39] and optimization based algorithms such as VINS-Mono [15] and OKVIS [40]. Tightly coupled methods joint all the IMU and camera states together and usually get more accurate results. Most of the VINS are developed to deploy on UAVs or handheld applications. They only fuse a single camera with IMU, as this already gives good results in their application. However, in optimization based algorithms, it is easy to add other residual terms, hence, making it possible to fuse IMU with stereo or even more other sensors [41]. This can also solve the problem mentioned in [21], which is that the scale is unobservable under special motions. However, they only give the results of 2D performance, and also requite a complex configuration of the stereo camera with the IMU sensor, especially w.r.t time synchronization. Besides the unobservability problem, initial values of VINS are crucial for the subsequent VIO (Visual Inertial Odometry) process, and lots of work [42,43,44] has been put into achieving better performance, mostly for handheld or UAV applications.

Combining RGBD sensors with IMUs for visual SLAM is another way to solve the scale unobservability problem [21], which is also suitable for ground robots navigation. RGBD sensors calculate the depth independently and efficiently and also output dense depth images rapidly. An IMU RGBD extrinsic calibration is proposed in [45], where they analyze the observability and design an OC (Observability Constrained) EKF to improve consistency. A loosely coupled method, which fuses inertial and RGBD cameras for mobile devices is introduced in [46], where an array of Kalman filters is used. Another loosely coupled method is proposed in [47], with application in direct methods of frame-to-frame motion estimation. There, the performance of the IMU aid is evaluated in both semi-dense and fully dense tracking processes. Ref. [48] is the first tightly coupled optimization-based method in RGBD inertial fusion, which focuses on 3D reconstruction with running in real-time on a GPU, while [49] is another extended Kalman filter based indoor scene reconstruction RGBD-inertial method. By utilizing inertial fusion and kernel cross-correlators to match point clouds in the frequency domain, Wang et al. [50] propose a non-iterative odometry, where a dense indoor fused map is given. Ling et al. [51] simply combine Kinect depth images with ORB features to achieve indoor 2D robot pose estimation. However, in this paper only limited qualitative results are reported.

Three-dimesnional reconstruction differs from mapping for robotics mainly in the fact that robots are primarily interested whether a location is occupied or free, while 3D reconstruction cares more about the texture of the estimated shapes. In robotics the original point cloud map is then usually treated as an input to different kinds of post-processes, that are employed to make use of the map. Elevation maps [52] are 2.5D representations which store one height-value per cell in the x-y plane. Multi-Level surface maps [53] are another more powerful map representation that also supports loop closing. OctoMap [54] is a real 3D map representation based on octrees, which can be dynamically expanded with regard to global and local planning scenes. Yang et al. [55] propose an update strategy to OctoMap after loop closing, which builds a globally consistent map.

A benchmark comparison of the VINS algorithms is discussed in [56]. Although it is aiming for flying robots, the results show that VINS-Mono [15] achieves the most accurate performance while having a low computational resource requirement. Therefore we build our the RGBD inertia system for 3D pose estimation of ground robots on the open source VINS Implementation and call it VINS-RGBD. We also extend the VINS system to include a mapping module that is making use of the dense depth information from the RGBD sensor. This 3D map enables further applications such as path planning and navigation. Hence, we propose to generate noise-eliminated point clouds in an octree.

## 3. VINS-Mono Analysis

**Figure 1:**
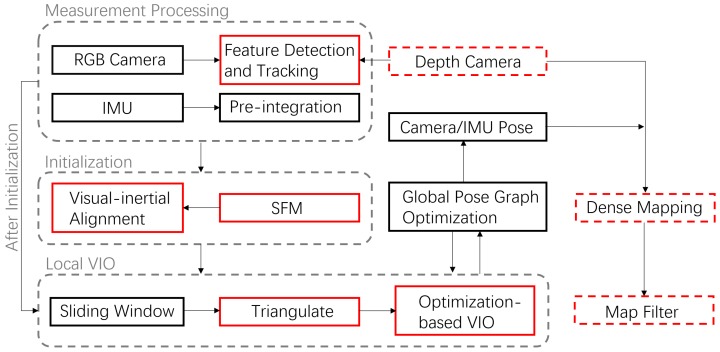
Core system structure. The solid line blocks indicate the original core pipeline of VINS-Mono. Red solid line blocks represent the modules of VINS-Mono that we modified. Red dashed line blocks represent the modules we newly added. VINS-Mono starts with the input of a color image sequence and IMU flow. IMU values are pre-integrated between every color frame, while visual features are detected and tracked. Then, the data is passed to SFM (Structure From Motion) to get an up-to-scale pose estimation. Once the visual and inertial alignment and initialization are done, the system starts to do a sliding window based local VIO process and is using the global pose graph optimization module to achieve the loop-closure task. The camera pose is then in a global consistent configuration.

VINS-Mono [15] is a tightly coupled, nonlinear optimization based method consisting of a feature tracking module, a visual-inertial odometry which contains the necessary estimator initialization process for the IMU parameters and the metric scale initialization, and a sliding window optimization method which is taking the initialization value as the start value. Moreover, a pose graph module is taking care of relocalization (i.e., loop detection) and the subsequent global optimization. It is a state-of-the-art visual-inertial system, which has a good performance on UAVs and hand-held applications. The system structure is shown in Figure 1. The red solid line blocks represent the modules we modified to incorporate depth information, and the red dashed line blocks are the modules we newly added. Black blocks are original modules of VINS-Mono. We first introduce the original blocks as the base of our work.

### 3.1. Vision Front End

VINS-Mono supports both rolling and global shutter cameras. It is using the KLT sparse optical flow algorithm [57] to track Shi–Tomasi features [58]. The features are made to be uniformly distributed over the images to achieve a robust tracking performance. Keyframes are selected by checking the average parallax between the current frame and the latest keyframes, along with the number of tracked features. If the parallax is beyond a certain threshold or the number of tracked features is below a certain threshold, a new keyframe is inserted.

### 3.2. IMU Pre-Integration

An IMU typically includes three orthogonal axis accelerometers ($a_{t}$) and a 3-axis gyroscope ($\omega_{t}$), which measure acceleration and angular velocity with respect to an inertial frame. Affected by noise, IMU measurements commonly contains additive white noise n and time-varying bias b:

(1) $$\begin{array}{c} {\tilde{a}}_{t}=a_{t}+b_{a_{t}}+R_{w}^{t}g^{w}+n_{a}\\ {\tilde{\omega }}_{t}=\omega_{t}+b_{w_{t}}+n_{w} \end{array}$$

As shown in Equation (1), accelerometers and gyroscopes suffer from noise that is usually modeled as Gaussian white noise,

(2) $$n_{a}∼N\left(0,\sigma_{a}^{2}\right),n_{\omega }∼N\left(0,\sigma_{\omega }^{2}\right)$$

The bias is usually modeled as a random walk process [15,17,59].

(3) $${\dot{b}}_{a_{t}}∼N\left(0,\sigma_{b_{a}}^{2}\right),{\dot{b}}_{\omega_{t}}∼N\left(0,\sigma_{b_{\omega }}^{2}\right)$$

The accelerometer estimation also suffers the need to compensate the gravity vector $g^{w}$ in world coordinate system. Earth’s rotation is usually neglected, because it is very small compared to the noise mentioned above.

MEMS-IMUs typically have an accelerometer and gyroscope output rate of 200–400 Hz, while cameras usually have a frame rate around 30 Hz. Since they are in different orders of magnitude, i.e., there are about 10 IMU measurements between two camera frames, IMU measurements are usually pre-integrated between two visual frames. The pre-integrated values are treated as constraints between the two camera frames, which is used later during the local window optimization together with the camera reprojection residuals.

We are using $b_{k}$ and $b_{k+1}$ to indicate two consecutive camera frames, where *k* is the frame index. As mentioned above, there are a couple of IMU measurements between time $t_{k}$ (at frame $b_{k}$) and time $t_{k+1}$ (at frame $b_{k+1}$). The pre-integration is then defined as follows:

(4) $$\begin{array}{cc} \alpha_{b_{k+1}}^{b_{k}} & ={\;\int \int \;}_{t\in \left[t_{k},t_{k+1}\right]}R_{t}^{b_{k}}\cdot a_{t}\;dt^{2}\\ \beta_{b_{k+1}}^{b_{k}} & =\int_{t\in \left[t_{k},t_{k+1}\right]}R_{t}^{b_{k}}\cdot a_{t}\;dt\\ \gamma_{b_{k+1}}^{b_{k}} & =\int_{t\in \left[t_{k},t_{k+1}\right]}\frac{1}{2}\Omega \left(\omega_{t}\right)\cdot \gamma_{t}^{b_{k}}dt \end{array}$$

The three items in Equation (4) α,β,γ are corresponding to translation, velocity and rotation, respectively. $a_{t}$ and $\omega_{t}$ are ideal values without noise and bias. Having $\omega^{\wedge }$ as the skew symmetric matrix of a vector, $\Omega \left(\cdot \right)$ is defined as follows:

(5) $$\Omega \left(\omega \right)=\left[\begin{array}{cc} -\omega^{\wedge } & \omega \\ -\omega^{T} & 0 \end{array}\right]$$

To save computational resources, the bias $b_{a_{t}}$ and $b_{\omega_{t}}$ are assumed to be constant during the pre-integration calculation between two consecutive camera frames. Then, the Jacobian matrixes with respect to every pre-integration items are calculated to correct the effect of bias changes. And if the estimated bias changes a lot, the pre-integration process is re-propagated under the new bias value.

### 3.3. Visual-Inertial Initialization and VIO

VINS-Mono tightly couples camera and IMU states in estimation. Also, as we have seen above, the IMU has a complex parameterization of the noise and bias. Thus, an initial process is necessary for parameter initialization of values such as gyroscope bias, gravity vector direction, and the metric scale for the up-to-scale mono visual SFM part. Once the initialization is done, the system can do the sliding window based local VIO process.

#### 3.3.1. Visual-Inertial Initialization

The SfM problem can be solved up-to-scale using only visual information. If the extrinsic $\left(p_{c}^{b},q_{c}^{b}\right)$, where ${\left(\cdot \right)}_{c}^{b}$ indicates translation (position p) or rotation (quaternion q) of the body/IMU frame with respect to the camera frame, is provided offline [60,61], then the IMU pose $\left(p_{b_{k}}^{w},q_{b_{k}}^{w}\right)$ is also determined up-to-scale by a scalar *s* and camera pose $\left(p_{c_{k}}^{w},q_{c_{k}}^{w}\right)$,

(6) $$\begin{array}{cc} q_{b_{k}}^{w} & =q_{c_{k}}^{w}⊗{\left(q_{c_{k}}^{b_{k}}\right)}^{-1}\\ sp_{b_{k}}^{w} & =sp_{c_{k}}^{w}-R_{b_{k}}^{w}p_{c_{k}}^{b_{k}} \end{array}$$

Here, *w* is set to equal the first camera frame, and ⊗ represents quaternions multiplication.

Combining these states with the IMU pre-integration term γ, we can calibrate the gyroscope bias. We have all IMU orientations $q_{b_{k}}^{w}$ and the pre-integration term γ in the window. Using B to represent all frame indexes in the window, the gyroscope bias is obtained by minimizing a least-square function:

(7) $$\begin{array}{c} \min_{\delta b_{w}}\sum_{k\in B}{∥{q_{b_{k+1}}^{w}}^{-1}⊗q_{b_{k}}^{w}⊗\gamma_{b_{k+1}}^{b_{k}}∥}^{2}\\ \gamma_{b_{k+1}}^{b_{k}}\approx {\hat{\gamma }}_{b_{k+1}}^{b_{k}}⊗\left[\begin{array}{c} 1\\ \frac{1}{2}J_{b_{\omega }}^{\gamma }\delta b_{\omega } \end{array}\right] \end{array}$$

Here $J_{b_{\omega }}^{\gamma }$ represents the Jacobian matrix of the pre-integration term γ with respect to $b_{\omega }$.

Combining the states with the IMU pre-integration terms α and β, the velocity, gravity vector, and scale can also be calculated by formulating a linear measurement model and solving this least square problem. We present this part in Section 4.2.

#### 3.3.2. Visual-Inertial Odometry

Once the initialization is done, the sliding window based local VIO process is active to continuously estimate the state. With extrinsic transform between the camera and IMU $\left(p_{c}^{b},q_{c}^{b}\right)$ calibrated offline, the state variables in the sliding window are defined as follows:

(8) $$\begin{array}{cc} X & =\left[x_{0},x_{1},\ldots x_{n},\rho_{0},\rho_{1},\ldots \rho_{m}\right]\\ x_{k} & =\left[p_{b_{k}}^{w},v_{b_{k}}^{w},q_{b_{k}}^{w},b_{a},b_{\omega }\right],k\in \left[0,n\right] \end{array}$$

*n* is the number of frames in the sliding window, $x_{k}$ is IMU state synchronized with the *k*th camera frame, which contains velocity, orientation and position, acceleration and gyroscope bias with respect to world frame. $\left[\rho_{1},\ldots \rho_{m}\right]$ represents the inverse depth from triangulation of all landmarks [62] in the frame of their first observation.

The IMU residual $r_{B}$ between two consecutive frame observations ${\tilde{z}}_{b_{k+1}}^{b_{k}}$ and states X is then defined(denoted as symbol ≐) as follows:

(9) $$r_{B}\left({\tilde{z}}_{b_{k+1}}^{b_{k}},X\right)≐\left[\begin{array}{c} \delta \alpha_{b_{k+1}}^{b_{k}}\\ \delta \beta_{b_{k+1}}^{b_{k}}\\ \delta \theta_{b_{k+1}}^{b_{k}}\\ \delta b_{a}\\ \delta b_{\omega } \end{array}\right]=\left[\begin{array}{c} R_{w}^{b_{k}}\left(\frac{1}{2}g^{w}\Delta t_{k}^{2}-v_{b_{k}}^{w}\Delta t_{k}+p_{b_{k+1}}^{w}-p_{b_{k}}^{w}\right)-{\tilde{\alpha }}_{b_{k+1}}^{b_{k}}\\ R_{w}^{b_{k}}\left(g^{w}\Delta t_{k}+v_{b_{k+1}}^{w}-v_{b_{k}}^{w}\right)-{\tilde{\beta }}_{b_{k+1}}^{b_{k}}\\ 2{\left[q_{b_{k}}^{w^{-1}}⊗q_{b_{k+1}}^{w}⊗{\left({\tilde{\gamma }}_{b_{k+1}}^{b_{k}}\right)}^{-1}\right]}_{xyz}\\ b_{a\;b_{k+1}}-b_{a\;b_{k}}\\ b_{\omega \;b_{k+1}}-b_{\omega \;b_{k}} \end{array}\right]$$

Here, $\delta \alpha_{b_{k+1}}^{b_{k}}$, $\delta \beta_{b_{k+1}}^{b_{k}}$ corresponds to translation and velocity IMU pre-integration residual terms, respectively. $\delta \theta_{b_{k+1}}^{b_{k}}$ is in axis-angle representation and corresponds to the rotation pre-integration residual term and is also the rotation residual. ${\left[\cdot \right]}_{xyz}$ is the function that converts quaternions to the axis-angle vector representation.

The camera visual residual $r_{C}$ is defined as the sum of the reprojection errors of every landmark visible in the window. Knowing the extrinsic calibration $\left(p_{c}^{b},q_{c}^{b}\right)$, the camera poses can be transformed to the IMU poses. Combined with all landmark depths in the state vector, it is easy to formulate the reprojection residual. During the sliding window based VIO process, new states are continuous added while old states are eliminated. Directly discarding the old states will cause a loss of accuracy, thus a marginalization strategy, implemented using the Schur complement [63], is used. The VIO optimization function containing the residuals of marginalization $r_{p}$, IMU $r_{B}$ and camera $r_{C}$ is written as:

(10) $$\min_{X}\left\{{∥r_{p}-H_{p}X∥}^{2}+\sum_{k\in B}∥r_{B}\left({\tilde{z}}_{b_{k+1}}^{b_{k}},X\right)∥+\sum_{(l,k)\in C}\rho \left(∥r_{C}\left({\tilde{z}}_{l}^{c_{k}},X\right)∥\right)\right\}$$

Here, $r_{p},H_{p}$ are the constructed prior information coming from the marginalization. The pair (l,k) indicates the *l*th feature observed in the *k*th camera frame in the window. ρ(·) is the Huber norm loss function to reduce outlier influence.

### 3.4. Loop Detection and Pose Graph Optimization

As the sliding window based VIO is running, the error is unavoidably accumulated, due to all kinds of noise, such as IMU and camera measurement noise, calculation accuracy error, the residual error at the end of the optimization as well as errors induced by the marginalization process. Like most SLAM systems, VINS-Mono also incorporates a loop closure model, which is using a Bag of Words approach (DBoW2) [32] for loop detection. Once the loop is detected, the loop constraint is added together with other relative transformation constraints between sequential keyframes. Then, the pose graph optimization process is active to distribute the error on all poses. In normal applications such as handheld and UAVs, the scale is observable thanks to the accelerometer measurements. The roll and pitch angles are also observable from the IMU. The global pose graph optimization hence, only needs to perform in 4-DOF of x,y,z, and yaw.

### 3.5. Observability Analysis of VINS-Mono

For dynamic systems, observability is a fundamental property, and the nullspace of the observability matrix is corresponding to system’s unobservable directions. More specifically, the rank of the nullspace is equal to the number of unobservable directions, while each unobservable direction has a corresponding physical meaning [64]. Besides, the observability is a system inherent property and hence, independent from the implementation. Since VINS-Mono is still using the normal VINS system modeling equations [64], it suffers from four unobservable directions, which are the 3-DOF global translation and the 1-DOF rotation around the gravity vector, which is the yaw angle. It is degenerated under special motions, which are frequently encountered with ground robots. For instance, when robots are moving at constant local acceleration,

(11) $$a_{t}^{b}≐R_{w}^{b}a_{t}^{w}\equiv a^{b},\;\forall t\geq t_{0}$$

such as a uniform linear motion of turning at a constant velocity, the system has another unobservable direction, which corresponds to scale. Note that the acceleration of the IMU is inherently measured in body frame, however to avoid ambiguity, we explicitly label the frame in Equation (11). To simplify the problem, we consider one state and change the corresponding visible landmarks expression in Equation (8) to contain x,y,z, denoted as *f*:

(12) $$Y=\left[p_{b_{k}}^{w},v_{b_{k}}^{w},q_{b_{k}}^{w},b_{a},b_{\omega },f_{0},\ldots f_{l}\right]$$

Thus, the corresponding up-to-scale state vector $Y^{\prime }$ can be written as:

(13) $$\begin{array}{cc} {p_{b_{k}}^{w}}^{\prime } & =sp_{b_{k}}^{w}\;=\;p_{b_{k}}^{w}+\left(s-1\right)p_{b_{k}}^{w}\\ {v_{b_{k}}^{w}}^{\prime } & =sv_{b_{k}}^{w}\;=\;v_{b_{k}}^{w}+\left(s-1\right)v_{b_{k}}^{w}\\ {q_{b_{k}}^{w}}^{\prime } & =\;q_{b_{k}}^{w}\\ b_{a}^{\prime } & =b_{a}-\left(s-1\right)b_{a}\\ b_{\omega }^{\prime } & =\;b_{\omega }\\ f_{i}^{\prime } & =sf_{i}\;=f_{i}+\left(s-1\right)f_{i},\;i=0,\ldots l \end{array}$$

where we use superscript ′ to represent up-to-scale. The error state [64] of $Y^{\prime }$ is therefore:

(14) $$\left[\begin{array}{c} {\left[\delta {q_{b_{k}}^{w}}^{\prime }\right]}_{xyz}\\ \delta b_{\omega }^{\prime }\\ \delta {v_{b_{k}}^{w}}^{\prime }\\ \delta b_{a}^{\prime }\\ \delta {p_{b_{k}}^{w}}^{\prime }\\ \delta f_{0}^{\prime }\\ \vdots \\ \delta f_{l}^{\prime } \end{array}\right]=\left[\begin{array}{c} {\left[\delta q_{b_{k}}^{w}\right]}_{xyz}\\ \delta b_{\omega }\\ \delta v_{b_{k}}^{w}+\left(s-1\right)v_{b_{k}}^{w}\\ \delta b_{a}-\left(s-1\right)a_{t}\\ \delta p_{b_{k}}^{w}+\left(s-1\right)p_{b_{k}}^{w}\\ \delta f_{0}+\left(s-1\right)f_{0}\\ \vdots \\ \delta f_{l}+\left(s-1\right)f_{l} \end{array}\right]=\left[\begin{array}{c} {\left[\delta q_{b_{k}}^{w}\right]}_{xyz}\\ \delta b_{\omega }\\ \delta v_{b_{k}}^{w}\\ \delta b_{a}\\ \delta p_{b_{k}}^{w}\\ \delta f_{0}\\ \vdots \\ \delta f_{l} \end{array}\right]+\left(s-1\right)\left[\begin{array}{c} 0_{3\times 1}\\ 0_{3\times 1}\\ v_{b_{k}}^{w}\\ -a_{t}\\ p_{b_{k}}^{w}\\ f_{0}\\ \vdots \\ f_{l} \end{array}\right]$$

After rewriting the equation, we get:

(15) $$\delta Y^{\prime }=\delta Y+\left(s-1\right)N_{s}$$

It is shown in [65] that $N_{s}$ lies in the nullspace of the observability matrix, under the condition that Equation (8) is satisfied. It is clear that the error state can be changed by arbitrary *s* with the same system measurements. Hence, the system is suffering scale unobservability under this special motion. The physical interpretation is also presented in [65]: The accelerometer bias can not be distinguished from true acceleration and since monocular VINS is relying on acceleration to achieve scale information, it is unobservable under this condition.

**Figure 2:**
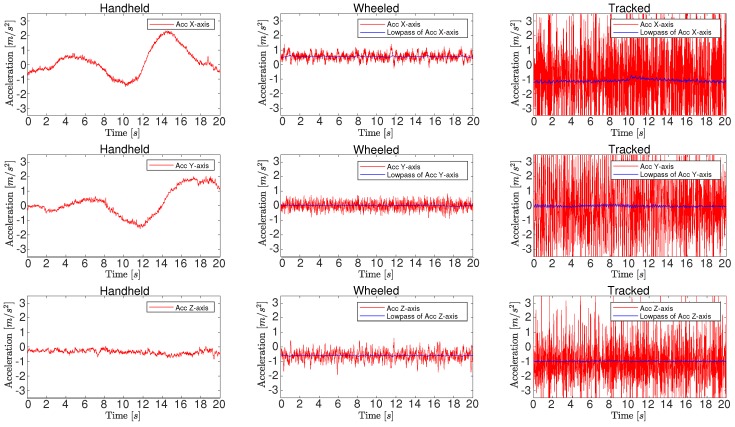
Acceleration values of handheld, wheeled robots and tracked robots shown separately. The left column is three-axis acceleration under normal handheld motion, the middle column is the acceleration of a wheeled robot which is driving a circular motion. The right column is the acceleration of a tracked robot which is under a uniform, linear motion. The red curves indicate measured raw acceleration values of the IMU sensors and the blue curves in the two right columns indicate the acceleration values after a lowpass filter.

To intuitively show the constant local acceleration motion, we plot measured acceleration values under three different scenarios in Figure 2. The columns from left to right represent handheld, wheeled robot under circular motions and tracked robot under uniform linear motion and rows from top to bottom represent the x,y,z axes separately. The raw acceleration values are plotted in red. Just for visualization in Figure 2 we also show the result of a lowpass filter in blue for the wheeled and tracked robot data, which contain lots of noise. It is clear that in the handheld applications, the IMU is sufficiently excited, especially in x and y direction, while wheeled and tracked robots under circular and uniform linear motion have almost constant acceleration. Besides, they suffer considerably from noise, especially for the tracked robots. Although IMU-preintegration can overcome most noise since if it is zero mean, the monocular system still becomes unstable when the noise is quite large, which is the usual condition of tracked robots.

On the other hand, when robots are performing no rotation motion

(16) $$R_{w}^{b_{t}}\equiv R_{w}^{b},\;\forall t\geq t_{0}$$

they suffer other unobservable directions, which makes all three global orientations unobservable. Wu et al. [21] developed mVINS (VINS within a Manifold), which incorporates VINS with manifold motions to solve the problem. Unfortunately, for rescue robots, the assumption is invalid due to the complexity of the terrain. Thanks to the loop closure model and our map filter, though it is left with an unobservable global orientation, we can still estimate a good trajectory and build nice maps.

## 4. VINS-RGBD

To address the scale unobservability problem described above and to improve the overall accuracy and robustness, we propose to incorporate the depth measurements of the RGBD sensor into the VINS-Mono system. We will be using the Realsense D435i RGBD sensor, which has an integrated IMU. Our algorithm contains a feature tracker node which is tracking features on the RGB image and gets the distance information from depth image. By utilize depth images, the system initialization gains another source of scale information, thus can be more robust and accurate. The VIO process is also working with the depth source to address the scale unobservable problem under constant local acceleration motion, but it also increases the accuracy for normal motions. Last but not least, we develop a mapping function which builds dense point clouds and eliminates noise at the same time.

### 4.1. Depth Estimation

The feature extracting and tracking method used as well as keyframe decision criteria are introduced as presented in Section 3.1. The tracked 2D features in the color images are combined with the pixel values in the same location of the depth images, which are aligned to the color frame. Then, the 3D points together with their coordinates in the image frame are passed to the following initialization or VIO process. Since the D435i sensor is using stereo infrared cameras with additional structure light, and also due to the intrinsic characteristic of depth sensor, holes and some patterns (zero measurement) of structure light remain, although the depth images have been processed by the hole-filling filter RealSense provides. On the one hand, if a 3D feature is observed multiple times in different frames, we can reject zero and other incorrect depth measurements with our depth verification algorithm. Points which cannot achieve relatively accurate depth are further passed to the original VINS-Mono triangularization process, where their depth can be estimated, as long as they are observed in two different color frames.

### 4.2. System Initialization

**Figure 3:**
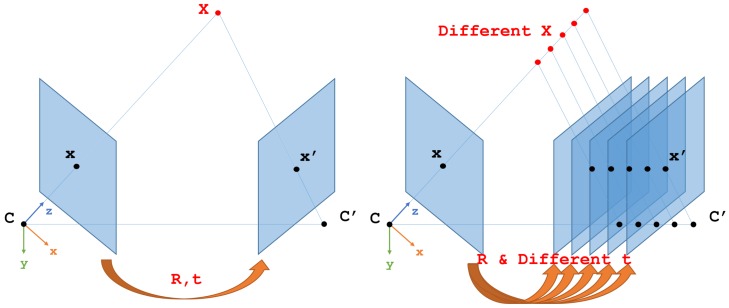
Illustration of 3D point projections on 2D images. *C* and $C^{\prime }$ are camera centers at different times with a rotation and translation *R*, *t* between them. *X* is a 3D point. *X*, *R* and *t* are unknown in monocular VINS. In the left image RGBD is used and the depth of the pixels is used to get *X* and PNP [67,68,69] is used to get *R*, *t* with constant scale factor. The right image illustrates the unknown scale factor when using just RGB. By having at least 5 point pairs like *x* and $x^{\prime }$, five-point algorithm [66] can be applied to get solutions.

After the tracking module, 3d points are extracted from each image with respect to the image frame which first observed them. VINS-Mono is using a window based optimization. We first need to set the value of the state vector, i.e., Equation (8). In VINS-Mono, since only the color source is available, the initial poses of the camera, hence, the IMU in window, are solved using the five-point algorithm [66]. The leads to the up-to-scale problem. Although the up-to-scale poses are then aligned to the IMU to achieve absolute scale, since IMU are always suffering noise, especially in ground robot applications and on low-cost IMU, the results of alignment are not always good and stable. With the depth source available in our VINS-RGBD approach, it is first feasible to perform the absolute scale pose initialization of cameras using PnP (Perspective-n-Point) algorithms [67,68,69]. The PNP problem is estimating the camera poses by using 2D features in the images and their corresponding 3D points in space. The difference of five-point algorithm and PNP algorithms is illustrated in Figure 3.

Equation (6) is then rewritten without scale *s*,

(17) $$\begin{array}{cc} q_{b_{k}}^{w} & =q_{c_{k}}^{w}⊗{\left(q_{c_{k}}^{b_{k}}\right)}^{-1}\\ p_{b_{k}}^{w} & =p_{c_{k}}^{w}-R_{b_{k}}^{w}p_{c_{k}}^{b_{k}} \end{array}$$

As shown in Equation (7), by getting the IMU pre-integration term γ, the gyroscope bias is calibrated initially. Since the scale is known, later parameters initialization only contains velocities and gravity vector, which can be calculated by minimizing terms $\delta \alpha_{b_{k+1}}^{b_{k}}$, $\delta \beta_{b_{k+1}}^{b_{k}}$ of Equation (9) in window with respect to variables $v_{b_{k}}^{w},g^{w}$. Thus, the state vector can be defined as:

(18) $$X_{k_{init}}=\left[v_{b_{k}}^{w},v_{b_{k+1}}^{w},g^{w}\right],k\in \left[0,n-1\right]$$

The linear measurement model is:

(19) $${\tilde{z}}_{k}=\left[\begin{array}{c} {\tilde{\alpha }}_{b_{k+1}}^{b_{k}}-R_{w}^{b_{k}}\left(p_{b_{k+1}}^{w}-p_{b_{k}}^{w}\right)\\ {\tilde{\beta }}_{b_{k+1}}^{b_{k}} \end{array}\right]=\left[\begin{array}{ccc} -R_{w}^{b_{k}}\Delta t_{k} & 0 & \frac{1}{2}R_{w}^{b_{k}}{\Delta t_{k}}^{2}\\ -R_{w}^{b_{k}} & R_{w}^{b_{k}} & R_{w}^{b_{k}}\Delta t_{k} \end{array}\right]≐H_{k}{X_{k_{init}}}^{T}+n_{k}$$

The linear least square problem is then defined by adding all the residual terms:

(20) $$\min_{X_{all_{init}}}\sum_{k\in [0,n-1]}{∥{\tilde{z}}_{k}-H_{k}{X_{k_{init}}}^{T}∥}^{2}$$

After solving the least square problem, another gravity refinement approach is applied as in original VINS-Mono, since the magnitude of the gravity is always known. By taking advantage of this information, the gravity vector can be modelled as a unit vector plus two DOF perturbation in its tangent space. Note that the acceleration bias does not include in the initialization process, since the gravity vector is a few orders of magnitude larger than the bias, which is making it hard to recover. This affects the scale recovery to a certain degree in VINS-Mono, however, has less influence on our VINS-RGBD.

### 4.3. Depth-Integrated VIO

The depth-integrated VIO process takes over the image streams once the initialization is done. The process start from the last state X in Equation (8). Then, the window slides once to marginalize the oldest or latest frame based on a parallax calculation, where keyframes are also decided. The next new $x_{k}$ is propagated by IMU integration and then added to the window. Based on all states in the window, in VINS-Mono points which do not have depth are triangulated to achieve depth. In our implementation, the depth is attached in each observation frame of a feature. Thus, a consistent depth verification Algorithm 1 can be applied to filter noisy depth measurements. For the points which are beyond the depth sensor’s range, the original triangularization method is used. Note that the sensor’s range does not need to be equal to device real range. It can be manually set by the user. This way the user can compensate for the increased error for faraway points in RGBD sensors.

**Figure 4:**
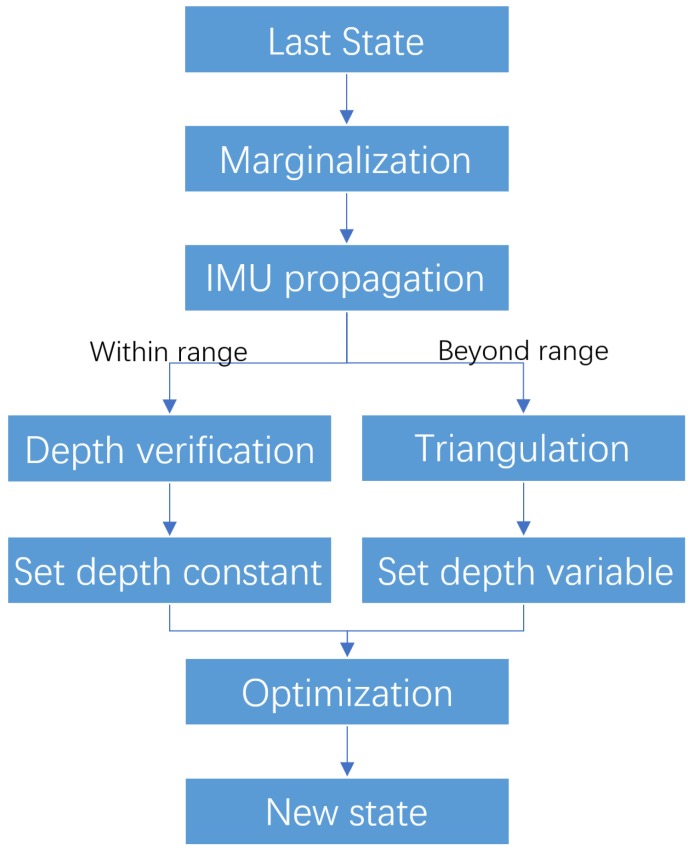
Depth integrated VIO process.

Once we have all features in the window with their verified depth, the camera visual residual, i.e., the reprojection error, can be calculated. With IMU residual and marginalization prior in Equation (10), the Ceres non-linear optimization solver [70] is used to solve the problem. However, for low-cost IMUs such as Realsense D435i is using, noise and bias are in bad condition, which violates the modelling in Equation (1). Hence, we set the features whose depth come from depth verification algorithms as constant while leaving the features whose depth come from triangularization still as optimization variables, as Figure 4 shows.

**Algorithm 1:** Depth verification algorithm.

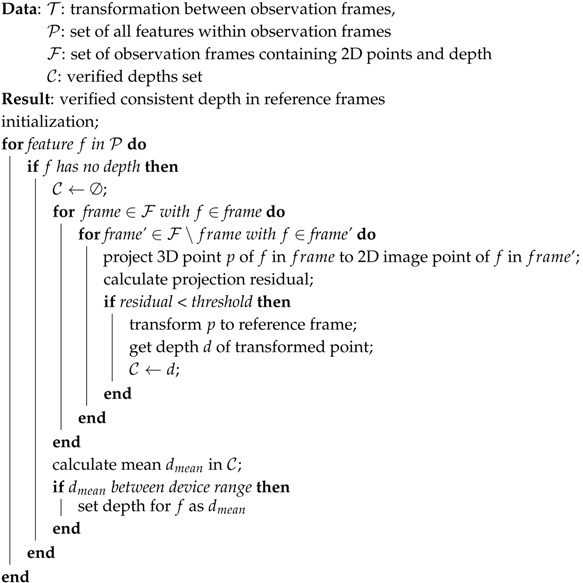



### 4.4. Loop Closing

After detecting a loop using the Bag of Words approach, VINS-Mono is estimating the transform between the two frames using a PnP approach on the 3D points estimated by triangulation. Thus, our VINS-RGBD is automatically also improving the loop closing transform estimation, since the used depths are now provided by the more reliable RGBD sensor.

### 4.5. Mapping and Noise Elimination

Different from Kinect Fusion [14], which uses GPU accelerated ICP [71] for finding matched points between two depth images, to make the system compact and suitable for small robot integration, we simply project 3D points from each depth image, which are synchronized with keyframes with their corresponding poses estimated by VINS, to build the map. With this straightforward method, however, it does not match corresponding points among frames, resulting in a lot of redundant points.

**Figure 5:**
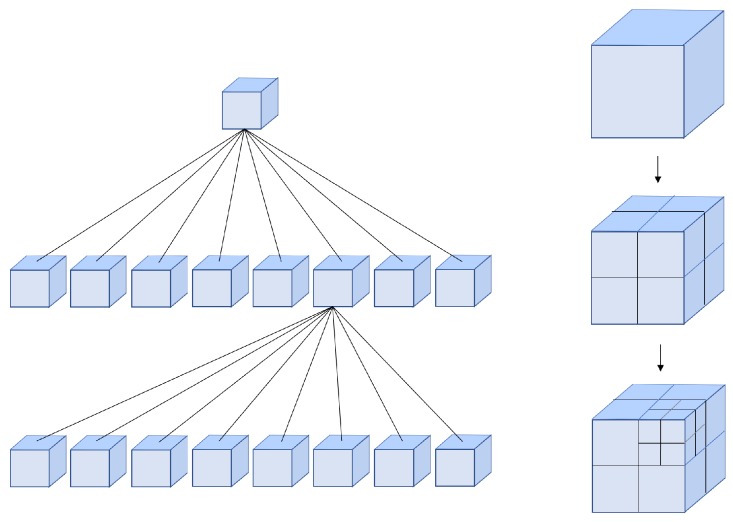
Octree Structure.

In order to better manage the point clouds, an octree [72] structure is maintained. Octree is a tree-like data structure used to describe three-dimensional space, each node of which represents a volume element of a cube. A parent cube can be at least cut into eight equal child cubes. Such the number of child nodes of any node in the tree cannot be anything but eight or zero. The volume elements represented by the eight child nodes are added together to be equal to the volume of the parent node. The resolution defines the size of the smallest voxel. A basic structure of octree is as shown in Figure 5.

To achieve real-time low-cost mapping and navigation, we first downsample every depth image with step size 10 to get around 3000 points, while the original depth image contains more than 300,000 points at a resolution of 640 × 480. Note that these points are inherently different from the feature points that the tracking thread provides. They are able to provide dense information, which is useful in navigation tasks, especially in the ground robot application, since they are not only doing obstacle avoidance but also interaction with obstacles in environments such as going over stairs.

**Figure 6:**
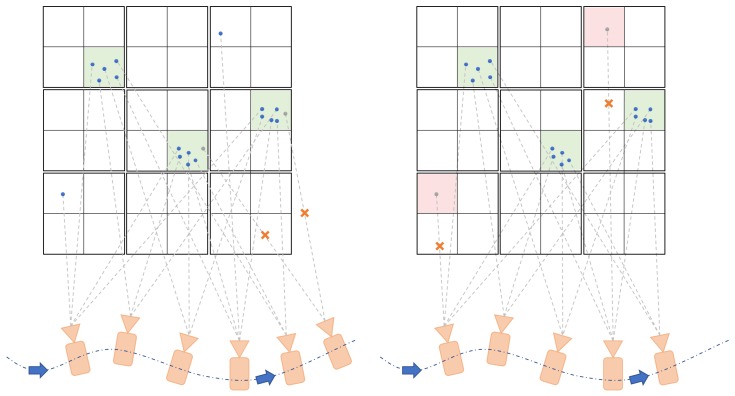
Points elimination. (**a**) illustrates the elimination during the VIO process. Cameras in figures represent keyframes. The points (blue) are continuously added to the octree leaf nodes until reaching the upper bound. Later coming points (gray) are rejected and also removed from point vector of corresponding keyframe to save space. After loop closure, as illustrated in (**b**), if a leaf node contains fewer points than a given threshold, the points are discarded, both in the octree and point vector of the keyframe.

After downsampling, we set the upper bound of each leaf node in the octree to only contain five map points. Any extra points coming later are rejected and also removed from the point vector attached on each keyframe, as Figure 6a shows. This may loss information, however, the resolution of the octree can always set high enough to satisfy the application demand. Also note that we are not aiming to do a 3D reconstruction, but to build a map applicable to ground robots, especially for rescue robot navigation.

On the other hand, when a loop is detected and closed, we need to update all the points after loop closure, where the time consumption is linearly related to the number of points. Setting the upper bound of the capacity of each leaf node significantly reduces the number of newly added points, ensuring the performance of real-time mapping. However, the loop closure algorithms minimize the error and thus may change all poses in the loop circle. There might still exist a few poses which have a significant error. The 3D points of those frames ideally should not be projected to the map. To achieve this we set a threshold on the number of points in a leaf: Points in leaves containing a fewer number than the threshold points are discarded. The threshold is depending on the velocity, frame rate and keyframe selection strategy. For instance, if the robots move fast, it may occur that most of the numbers of points in octree leaf nodes are less than the threshold and be discarded incorrectly. Besides, the depth image provided by the depth camera has noise which considered as random. Our elimination strategy can also discard them, thus leading to a more consistent map. The elimination process is shown in Figure 6b.

## 5. Experiments and Results

To our knowledge, there is no public dataset which contains color, depth and IMU data together. The EuRoC MAV visual-inertial datasets [73] contains stereo images rather than dense depth images directly. Besides, they are also aiming to MAV (Micro Aerial Vehicle) applications. The RGBD SLAM Datasets [74] provided by TUM contains RGBD images; however, they only have accelerometer data. To this end, the experiments are all evaluated in real-world environments compared to the original VINS-Mono by using a RealSense D435i camera. The datasets are also attached with our open source code. To comprehensively evaluate our system, we perform both single and integrated experiments. The first single experiment evaluates the scale drift problem during local constant acceleration motion. The second single experiment validates our depth-integrated VIO process illustrated in Figure 4. The integrated experiments with handheld, wheeled and tracked robots are then performed to show the versatility of our system. Last but not least, the mapping results are shown in different scenarios. The experiments are all running on an Intel NUC equipped with i7-8559U CPU at 2.7–4.5 GHz and 16 GB memory; no GPU is used. The ground truth poses in experiments are provided by our OptiTrack [75] tracking system and we evaluate the trajectories using the methods provided by [76].

### 5.1. Scale Drift Experiment

**Figure 7:**
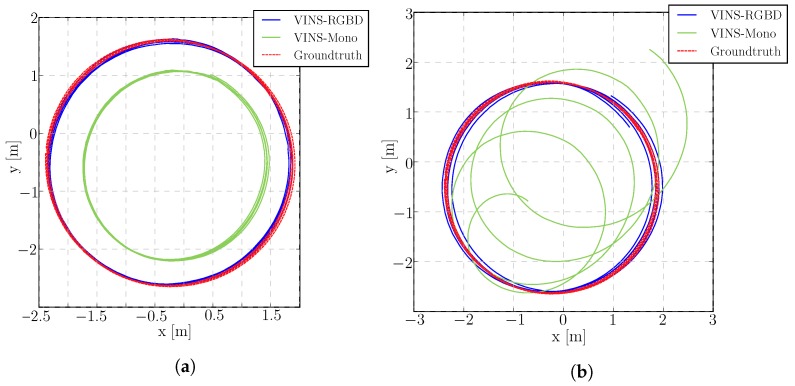
Scale drift evaluation with loop closure (**a**) and with deactivated loop closing (**b**). The trajectories are produced by a wheeled Jackal UGV.

**Figure 8:**
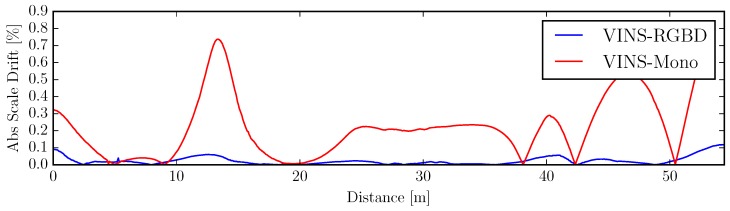
Absolute scale drift percentage of the deactivated loop closing experiment.

The scale drift experiment is performed on a Jackal [77] UGV, driving at a constant radius circle. When the monocular VINS system is under constant radius circular motion (radius about 2 m, speed about 0.4 m/s), the scale direction is unobservable, as we showed in Section 3.5. However, combined with absolute depth information, another scale resource is integrated into the system. Figure 7 illustrates our VINS-RGBD compared to VINS-Mono with ground truth. For comparison we also tested both systems with deactivated loop closing (no loop), showing the performance of only the pure VIO. Our system is well aligned with the ground truth in both loop and no loop conditions. Since RealSense D435i is using a low-cost IMU, the monocular VINS drifts a lot as shown in Figure 8. Actually, VINS-Mono initialized with a wrong scale, which can be seen in Figure 7b.

### 5.2. Open Outdoor Experiment

**Figure 9:**
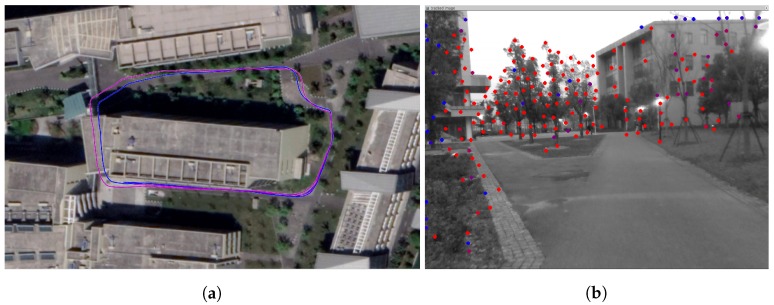
Open outdoor experiment. (**b**) shows a representative moment when our system is running, where the points are far away and most of them are beyond depth range of the device. The color in more red indicates the longer of tracking times. (**a**) shows the trajectories aligned to Google Maps. The blue one is estimated by our system, and the purple one is from VINS-Mono.

RGBD cameras usually use structured light or ToF (Time of Flight) technology to achieve dense depth images, which also limits the measurement range to several meters. Also, since they are active infrared sensors, they usually get over shined by the sunlight. For those reasons almost all handheld and UAV applications use color only VINS. RealSense D400 series is using a stereo approach with structure light as backup for uniform surfaces and has a maximum range up to 10 m. The error also increases exponentially with distance. In this paper we proposed to use a verified depth within a certain range and use the triangularization method to estimate points beyond depth measurement range, as shown in Figure 4. We evaluate this part in a handheld open outdoor scenario which shown in Figure 9b. The trajectories estimated by VINS-Mono and our system aligned to Google Maps are shown in Figure 9a. We can see the performance of both algorithms is similar, which validates that our system does not degenerate in outdoor and out-of-range scenarios.

### 5.3. Integrated Experiments

The integrated experiments cover handheld, wheeled robot and tracked robot tests. We evaluate the trajectories of our proposed VINS-RGBD and VINS-Mono with the ground truth poses from the tracking system. The RMSE (Root-Mean-Square Error) is used to evaluate the absolute error after aligning every trajectory to its corresponding ground truth path. The absolute translation and orientation are also calculated to compare. Besides, the relative pose error is shown to give a more comprehensively evaluation. To highlight the comparisons between the two methods, we disable the loop detection and optimization in the following tests, however, loop performance is also evaluated in the form of RMSE in Table 1.

#### 5.3.1. Handheld and Wheeled Experiment

**Figure 10:**
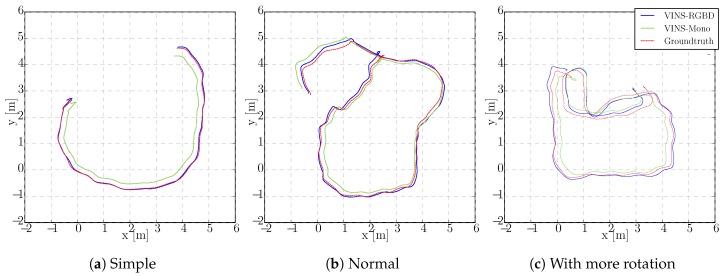
Handheld trajectory estimation.

**Figure 11:**
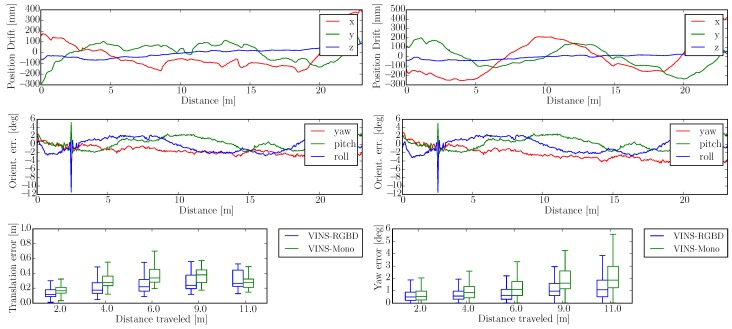
Handheld Absolute and Relative Error in “With more rotation” Experiment. The first two rows show the absolute position and orientation error, where the left results are from our VINS-RGBD and right are from VINS-Mono. The last row shows the relative translation and yaw error of our VINS-RGBD and VINS-Mono, respectively.

To show the versatility of our system, we perform handheld experiments with RealSense D435i under different motions. The trajectories are shown in Figure 10 and the absolute and relative error of the test “With more rotation” is shown in Figure 11. It is shown that with depth information integrated, the handheld application, which is like UAVs, also has better performance compared to the original VINS-Mono. In Figure 10a,c, the scale evaluation of VINS-Mono method is in bad condition, however, our method not only recovers scale more accurate but also has better performance in relative pose estimation, which is shown in relative error part of Figure 11.

**Figure 12:**
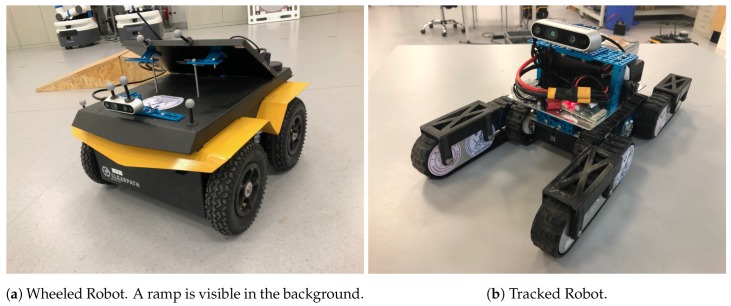
Robot Hardware Platform.

**Figure 13:**
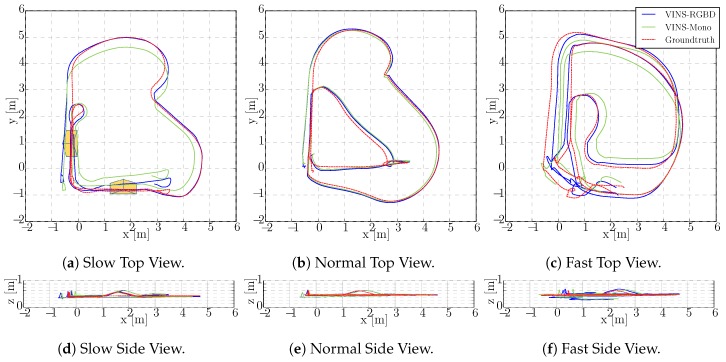
Wheeled Robot trajectory estimation. Driving on flat ground with two ramps (approximate position indicated in (**a**). (**b**,**c**) are similar).

**Figure 14:**
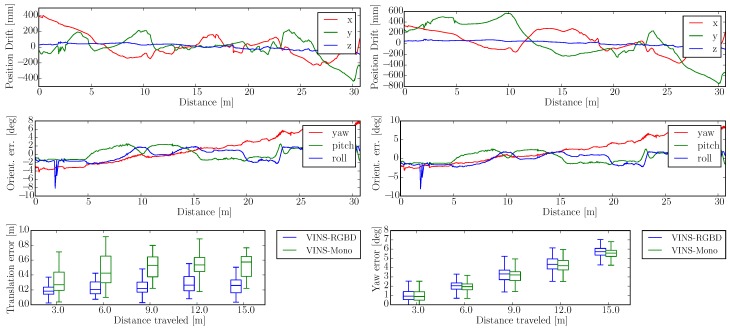
Wheeled Robot Absolute and Relative Error in “Slow” Experiment. The first two rows show the absolute position and orientation error, where the left results are from our VINS-RGBD and right are from VINS-Mono. The last row shows the relative translation and yaw error of our VINS-RGBD and VINS-Mono respectively.

The wheeled robot hardware platform is shown in Figure 12a, which is a commercial wheeled ground vehicle with differential drive. The wheeled acceleration reading of IMU in Figure 2 is from this platform, which is shown that small sized wheeled robots usually suffer larger acceleration noise than handheld applications. This is due to the mechanism of such robots, because large vehicles such as passenger vehicles usually have larger wheels which can absorb more shock and independent shock mitigation systems that small-sized robots do not have. In the rescue field, small sized wheeled robots are also used sometimes in easy terrain. Figure 13 and Figure 14 show the trajectories and error comparison of our wheeled robot. The trajectories are from the robot driving on the ground with two up-down slopes with different velocities (average speeds: slow: 31 min 148 s; normal: 32 min 74 s; fast: 48 min 99 s (with intermittent high speed phases)). Our VINS-RGBD aligned to ground truth better, especially in slow and fast experiments. In the normal velocity test, both methods have good performance, which may be caused by the sufficiently activated accelerometer under this motion. However, with faster motion, which is shown in Figure 13c, since more motion blur from the color camera is introduced, the performance degenerates. In the relative error diagrams of Figure 14, the relative translation error performance is much better in our method while relative yaw does not have much difference, which is as was expected since, adding depth information into VINS has a large effect on translation evaluation but little on orientation.

#### 5.3.2. Tracked Experiment

**Figure 15:**
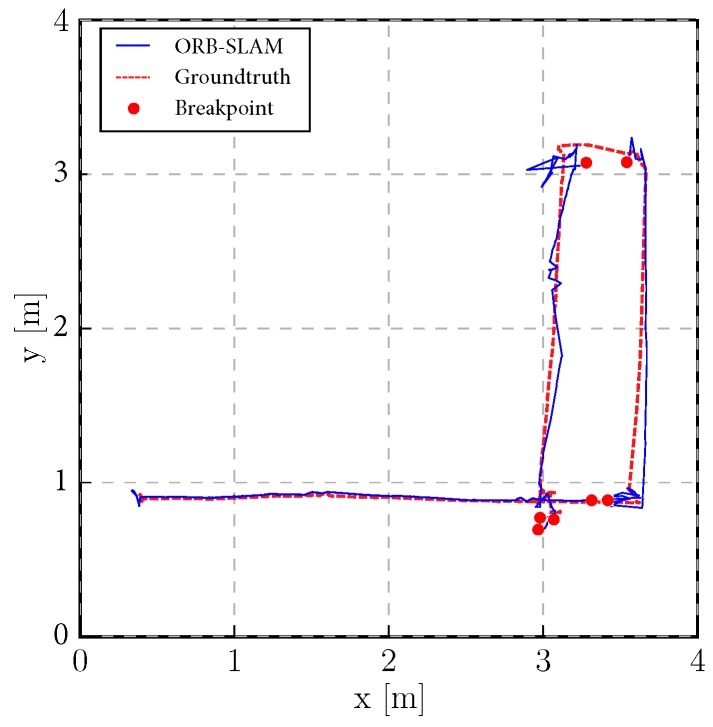
ORB-SLAM in Tracked 2 (Cross and Up-down Slopes) test. It lost track three times. We reset the ORB-SLAM once it lost track and get four individual trajectories. The red points indicate the beginning and end of four trajectories. Note that since we align trajectories separately, the aligned result could look good, which should not be treated as the real performance of this method.

**Figure 16:**
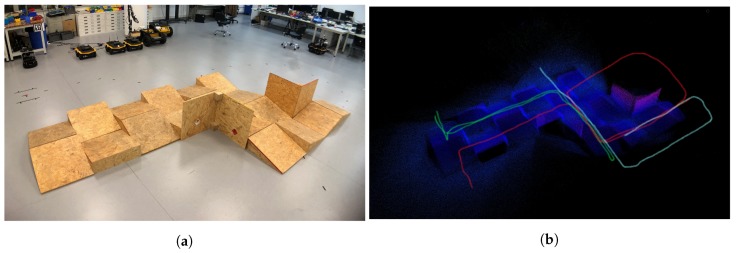
Tracked robot experiment site. (**a**) Real Trajectory Testing Environment; (**b**) Hand-drawn trajectories of the tracked robot experiments. Tracked 1: light-blue; Tracked 2: green; Tracked 3: red.

**Figure 17:**
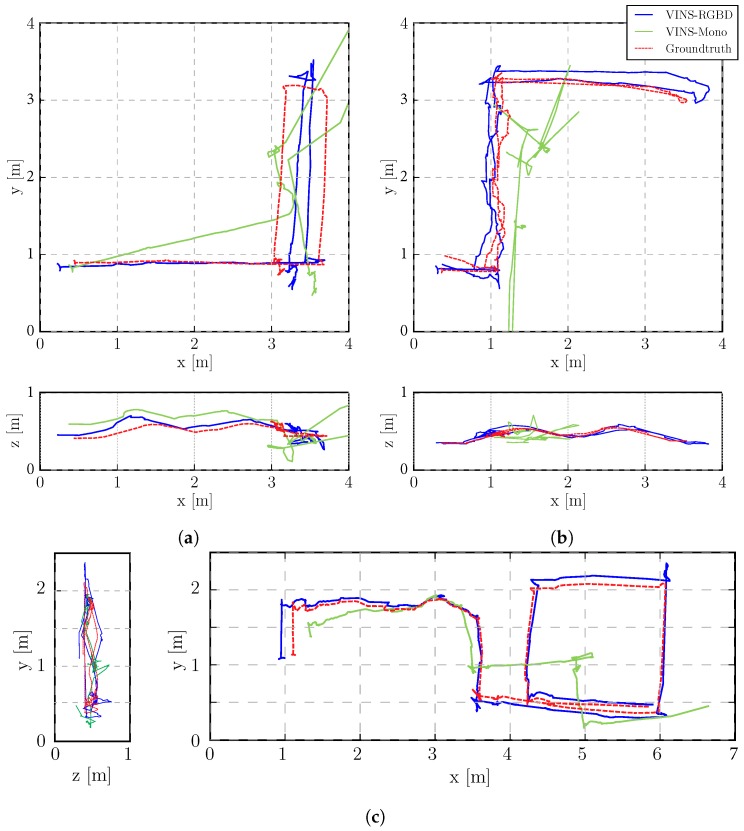
Tracked robot trajectory estimations. Start point on the left for all three experiments. (**a**) Tracked 1: Ground and Up-down Slopes. Top View (top), Side View (bottom); (**b**) Tracked 2: Cross and Up-down Slopes. Top View (top), Side View (bottom); (**c**) Tracked 3: Cross and Up-down Slopes and Ground. Top View (right), Side View (left).

**Figure 18:**
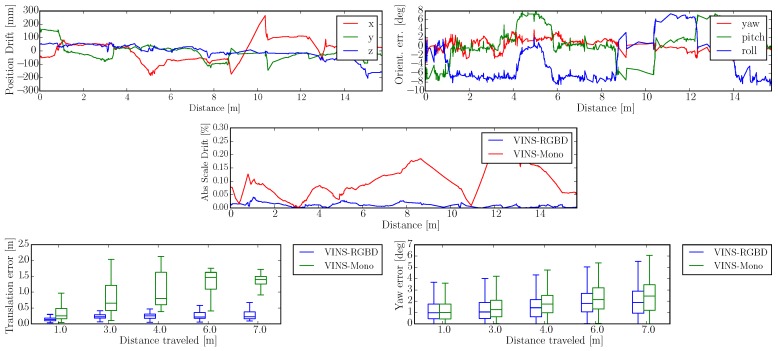
Tracked Robot Absolute and Relative Error in the “Cross and Up-down Slopes and Ground” experiment. The first row shows the absolute position and orientation error of our system. The relative translation and yaw error together with the scale drift percentage comparison of our VINS-RGBD and VINS-Mono are shown in the last two rows.

**Figure 19:**
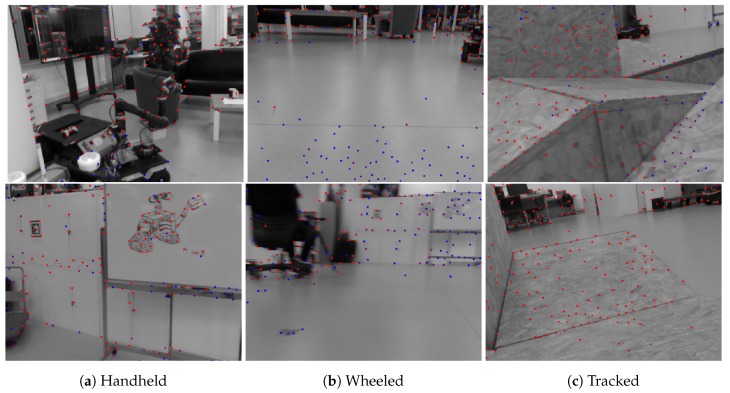
Selected frames from the handheld, wheeled and tracked experiments.

The self-developed tracked robot hardware platform is shown in Figure 12b, which has a typical operation size of 0.4 × 0.3 × 0.4 m, and four independent flippers for obstacle crossing. Since the size is smaller than regular rescue robots, there is lots of shaking caused by tracks during the movement, which causes pose estimation to be more difficult, as Figure 2 shows. Besides, the movement in 3D scenes usually contains sudden falls from obstacles, and fast rotations, which causes strong ego-motion and makes the camera only SLAM such as ORB-SLAM2 fail, as shown in Figure 15. We perform the tracked robot experiments in three different tests: Ground and Up-down Slopes; Cross and Up-down Slopes; Cross and Up-down Slopes and Ground. The schematic trajectories are shown in Figure 16, which is the resulting map from our method under the handheld approach, with light blue, green and red colors, respectively. The three trajectories evaluations are shown in Figure 17 and Figure 18. As can be seen in Figure 17, the trajectories are flexuose even on the ground truth. The performance degenerated due to this factor, where VINS-Mono does not work in the first two tests and also hardly works in the last test. Our method has a larger error than previous handheld and wheeled tests. However, it is still at an acceptable level. The RMSE of all the nine experiments is listed in Table 1, which shows that with depth-integration, our method has a better performance. Figure 19 shows representative selected frames in handheld, wheeled and tracked robots, respectively.

### 5.4. Map Comparison

In Section 4.5 we proposed to bound the point clouds by eliminating points using octree during the VIO process. The resulting point clouds are further filtered if an octree leaf node contains fewer points than another threshold. This strategy can build maps with different density point clouds, which also accelerates the loop closing speed.

The experiments are performed a 60 cm version of the ASTM Standard Test Method for Evaluating Emergency Response Robot Capabilities. Those are ASTM E2803-11 Confined Area Obstacles: Inclined Planes and ASTM 2827-11 Crossing Pitch/Roll Ramps [78], the first test methods with the rails is still in the standardization process. Those test methods are also extensively used in RoboCup Rescue [79,80], for which we plan to use VINS-RGBD.

**Figure 20:**
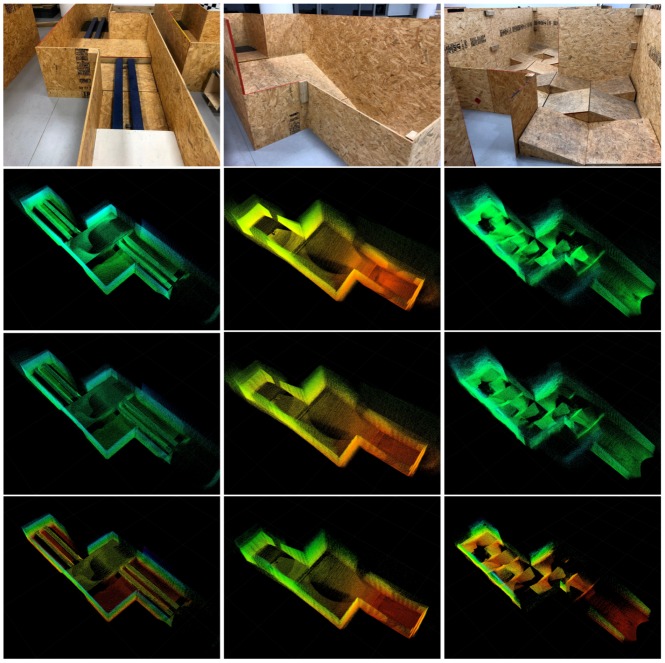
Mapping results in ASTM Standard Test Methods.

**Figure 21:**
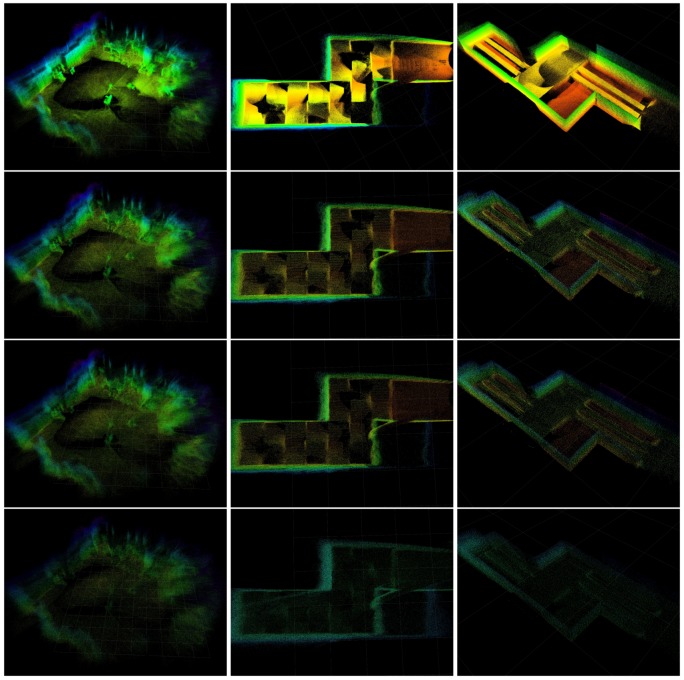
Mapping result corresponding to comparison in Table 2, from left to right: lab (large), arena (middle), arena (small).

Figure 20 shows the results of our VINS-RGBD mapping. In every column, there is one of the test methods. The rows with the maps show the mapping with all subsampled points on the top, the maps with the upper limit of five points in an octree cell in the middle and the maps where additionally points in cells with fewer than three points are filtered in the bottom. Note that the last filter step is triggered manually here to keep it consistent with the above maps. Comparing the third rows to the second rows, we can see that the map structure remains very similar while the point density is reduced. The last rows eliminate points if fewer than a threshold of 3 are in a cell. We can see that the point clouds is further reduced, mainly in areas which are of little interest. To quantitatively evaluate the efficiency of the octree structure based strategy, we perform experiments in three different size scenes: lab (large), arena (middle), and arena (small) in Table 2. The corresponding map results are shown in Figure 21. The experiments evaluate the upper bound number during the VIO process, filtering after loop closing is disabled to keep the results consistent. We can see with the upper bound number decreasing, the number of points is also reduced near linearly. The last two columns list the update time after loop closure with an octree leaf capacity at 5 and the speed up rate compared to origin points. This is calculated by points rate, since the points update time is linearly related to the number of points.

## 6. Conclusions

We proposed a system to fuse color, depth and inertial sensors for trajectory estimation and mapping for small ground rescue robots. Our system called VINS-RGBD is based on the state-of-the-art software VINS-Mono. We are taking advantage of the RealSense D435i, which is small, lightweight and has an integrated IMU. We extend the VINS-Mono system to make use of the depth data during the initialization process as well as during the VIO phase. Exhaustive experiments and comparisons with handheld, wheeled robots and tracked robots are performed to evaluate the performance and versatility of our system. Our software is available as open source and the dataset is also provided. The experiments show the excellent performance of our system compared to the ground truth trajectories, to the VINS-Mono system and also to an ORB-based approach. We show that for ground robots, especially for tracked robots, the noise of the IMU readings is very high due to the vibrations induced by the ground contact. More importantly, under some special motions common for ground robots, the acceleration bias cannot be estimated since it is unobservable. This is a problem for traditional VIO systems, that are relying heavily on the IMU to estimate the unknown scale factor. Using depth information we can prevent the IMU from having a too big negative effect on the results, thus enabling our solution to provide good maps for applications such as path planning or navigation.

In this work, we fuse the RGBD camera data with both gyroscope and accelerometer measurements from the IMU. However, in some extreme situations such as very challenging terrain, the accelerometer is susceptible to high-frequency noise and becomes unreliable. One naive way is to only fuse the RGBD camera images with the gyroscope and don’t use the accelerometer, since the depth camera already provides absolute scale information. However, both accelerometer and depth camera have their degeneration conditions. Using both accelerometer and depth cameras also has advantages, e.g., the gravity vector estimated from the accelerometer can correct the pitch and roll angle of the camera. Thus, we keep both and are working on finding better ways to model the accelerometer, especially in extreme conditions. With a pose ground truth, such as recorded in our dataset, it is easy to get accelerometer ground truth and make it possible to use deep learning to model the accelerometer or even IMU more correctly. With better sensors modelling, we expect to achieve more accurate trajectory estimation and mapping results.

To build a whole navigation system, we are also working on flipper planning of our tracked robot under the limited environmental perception, since RGBD cameras typically only provide one view of the environment rather than 360 degrees by LIDAR. The 3D path planning is also a further topic together with ground robot navigation map generation, by utilizing the consistent point cloud information provided by our VINS-RGBD system. 

## Figures and Tables

**Table 1:** RMSE of three different experiments in meters. “-” denotes no loop, “x” denotes test failed.

Experiment	VINS-RGBD	VINS-RGBD with Loop	VINS-Mono	VINS-Mono with Loop
Handheld Simple	**0.07**	-	0.24	-
Handheld Normal	**0.13**	-	0.20	-
Handheld With more Rotation	**0.18**	0.20	0.23	0.23
Wheeled Slow	0.20	**0.16**	0.39	0.27
Wheeled Normal	0.17	**0.09**	0.18	**0.09**
Wheeled Fast	0.23	**0.20**	0.63	0.31
Tracked 1 Ground and Up-down Slopes	0.11	**0.10**	x	x
Tracked 2 Cross and Up-down Slopes	**0.17**	0.23	x	x
Tracked 3 Cross and Up-down Slopes and Ground	**0.21**	0.24	0.77	0.75

**Table 2:** Efficiency of the octree structure.

Scene	Octree Resolution (m)	Origin (Points)	Octree 5pts	Octree 3pts	Octree 1pt	Loop Cost 5pts (ms)	Speed up (5pts)
lab (large)	0.05	3,981,242	2,026,884	1,562,519	760,411	378	49.1%
arena (middle)	0.02	6,153,656	849,748	619,237	284,029	159	86.2%
arena (small)	0.02	4,259,847	198,161	128,615	48,091	37	95.3%
